# MS-YOLOv11: A multi-scale feature fusion network for real-time rooftop photovoltaic detection from UAV images

**DOI:** 10.1371/journal.pone.0346424

**Published:** 2026-05-06

**Authors:** Jiajun Zhu, Hongxi Jin, Depeng Gao, Xiangxiang Mei, Shuxi Chen, Jianlin Qiu, Haifei Zhang

**Affiliations:** 1 College of Yonyou Digital Intelligence, Nantong Institute of Technology, Nantong, China; 2 School of Information Engineering, Nantong Institute of Technology, Nantong, China; Aalto University, FINLAND

## Abstract

With the rapid proliferation of distributed solar energy, Unmanned Aerial Vehicles (UAVs) have emerged as indispensable tools for rooftop photovoltaic (PV) inspection. However, practical deployment is hindered by hierarchical challenges, including extreme scale variations of PV targets, complex background interference, and the limited computational resources of edge devices. To address these issues, this study proposes a lightweight yet high-performance detection network based on the YOLOv11 architecture. First, to mitigate the missed detection of small-scale targets, a high-resolution P2 detection layer is constructed to preserve fine-grained features from shallow layers. Second, a Large Selective Kernel Attention (LSKBlock) mechanism is integrated into the backbone, enabling dynamic receptive field adjustment to enhance feature extraction capabilities in cluttered environments. Finally, the G3Ghost module is introduced to counterbalance the computational overhead of the multi-scale structure. This module leverages linear transformations to reconstruct redundant features, significantly compressing the model volume without compromising precision. Experimental results on a specialized UAV-based rooftop PV dataset demonstrate superior performance. Compared with the baseline model, the proposed method achieves a precision of 96.32%, a recall of 95.74%, and an mAP@0.5 of 98.5%. Notably, the model is exceptionally lightweight, with only 2.04 M parameters and a 4.5 MB storage footprint, while maintaining a high inference speed of 115 FPS. These metrics indicate that the proposed approach achieves an optimal trade-off between detection accuracy and operational efficiency, providing robust technical support for mobile deployment on UAV platforms.

## Introduction

The rapid expansion of urban distributed photovoltaic (PV) systems is evident. This has made automated asset management and capacity estimation pivotal links in achieving carbon neutrality goals [1]. However, rooftop environments are complex. Traditional manual inspections in these settings are time-consuming and labor-intensive. They are also prone to errors. Defect identification is a core requirement for PV operation and maintenance (O&M). Yet, achieving high-precision localization of PV panels from Unmanned Aerial Vehicle (UAV) imagery remains a technical bottleneck [2]. This is primarily attributed to drastic scale variations in aerial targets. Additionally, these targets face pervasive interference from complex backgrounds.

Early PV detection methodologies relied heavily on manual labor. They also depended on conventional physical inspection techniques. Electroluminescence (EL) imaging excels in identifying internal micro-cracks [3]. However, this technology requires the PV system to be in an energized state. This makes large-scale outdoor deployment impractical. Subsequently, traditional image processing techniques were widely adopted [4–5]. These include methods such as edge detection and threshold segmentation. Nevertheless, these methods exhibit insufficient robustness under complex backgrounds and fluctuating illumination. They fail to meet the rigorous demands of automated O&M [6].

Breakthroughs in deep learning have introduced transformative solutions for PV inspection. Early research primarily focused on two-stage detection algorithms. Faster R-CNN is a prime example [7]. For instance, Vlaminck et al. [8] utilized region-based CNNs. They successfully achieved defect identification in aerial PV imagery. Such algorithms offer high detection accuracy. However, their network structures are complex. This results in excessive inference latency. Consequently, they are difficult to deploy on UAV-mounted edge devices for real-time processing. Therefore, one-stage detection algorithms have emerged as the dominant research paradigm [9–11]. These are represented by the YOLO (You Only Look Once) series. They are favored for their superior balance between detection speed and accuracy. Almadhor et al. [12] validated the efficacy of AI-driven frameworks. Their work focused on early PV defect recognition. Subsequent research focused on refining YOLOv5. Li et al. [13] developed the GBH-YOLOv5 algorithm. It integrates Ghost convolutions and a tiny target prediction head. This effectively reduced the missed detection rate of small targets. Similarly, Wang et al. [14] and Zhang et al. [15] optimized YOLOv5. Their improvements enhanced surface defect detection performance.

Algorithms continue to evolve. YOLOv10 and YOLOv11 have demonstrated state-of-the-art performance [16–17]. Qu et al. [18] proposed a high-precision model based on YOLOv10. This model incorporates Compact Inverted Blocks and Partial Self-Attention modules. It can accurately identify “black core” defects and damaged areas on PV panels. Ghahremani et al. [19] conducted a systematic evaluation in a recent comparative study. They assessed YOLOv10 and YOLOv11. Results showed that YOLOv11 achieved a mean Average Precision (mAP) of 92.7% on thermal and optical datasets. This underscores its potential as a robust baseline model. Despite these advancements, YOLOv11 still faces severe challenges in rooftop PV detection. The primary issue is multi-scale feature misalignment. In UAV imagery, target scales vary drastically. Targets range from large occlusions to minute cracks. Furthermore, backgrounds are cluttered with HVAC units and various conduits. To address this, Zhang [20] introduced a multi-scale feature pyramid detection network. He emphasized the necessity of multi-scale feature fusion to minimize information loss. Additionally, Cai [21] recently refined YOLOv11n into the YOLO-PV model. This model utilizes an Enhanced Hybrid Multi-scale Block. It improved Precision by 6.7% and mAP@0.5 by 2.9%.

Practical deployment of UAV PV inspection follows a hierarchical logic. This logic is “localization-first, diagnosis-later.” Existing literature has extensively explored defect classification. However, system reliability is strictly constrained by the initial detection accuracy of PV panels. PV panels often appear as multi-scale small targets in dynamic urban settings. They are frequently obscured by shadows or adjacent structures. Failure in the initial localization phase leads to high false-alarm rates in subsequent diagnostics. This severely undermines the system’s utility. Therefore, developing a high-precision algorithm with multi-scale robustness is crucial. It is not merely a preliminary step for automated energy audits and O&M. It is a decisive factor for success. Accordingly, this paper proposes an improved YOLOv11 algorithm. It is based on multi-scale feature fusion. The goal is to enhance cross-scale feature interaction and achieve efficient, precise rooftop PV detection.

The primary contributions of this work are as follows:

1. Baseline Benchmarking: We conducted extensive comparative experiments. These validated YOLOv11 as the optimal baseline architecture for UAV-based rooftop PV detection.
2. Architectural Enhancement: We integrated a high-resolution P2 detection head and a Large Selective Kernel Attention module. This effectively resolved multi-scale feature misalignment. It also boosted detection performance in cluttered environments.
3. Lightweight Optimization: We incorporated a GhostNet-based feature extraction module. This significantly compressed the model volume. It provides robust technical support for deployment on edge devices and mobile platforms.

## Materials and methods

### Dataset description

The dataset employed in this study primarily consists of high-resolution remote sensing satellite imagery. [22–23]. The dataset covers varied geographical environments and lighting conditions. It also includes rooftop PV modules with differing dimensions and spatial layouts. All images were standardized to a resolution of 640 × 640 pixels. Crucially, we preserved the original spatial textures and fine-grained spectral features during this process.

The dataset underwent rigorous screening and organization. It comprises a total of 13,360 high-resolution rooftop PV images. Each image contains annotations for multiple detection targets. We divided the dataset to support systematic model development and performance evaluation. Following the COCO data format, we split the data into a training set, a validation set, and a main test set (T1). The allocation ratio was set at 8:1:1. The training set optimizes the network’s internal parameters and weights. The validation set allows for hyperparameter fine-tuning during training. It also serves to monitor potential overfitting risks. The main test set (T1) evaluates the improved model’s generalization performance on unseen data within the same domain.

**Fig 1:**
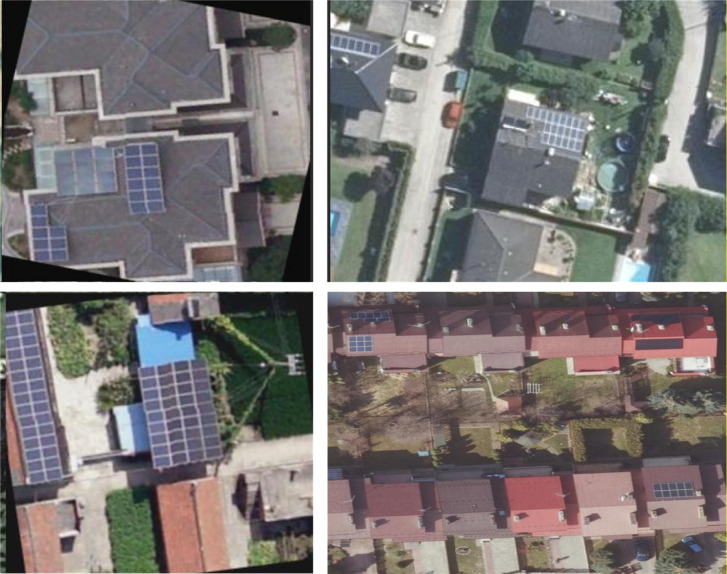
UAV – perspective photovoltaic panel dataset images.

**Fig 2:**
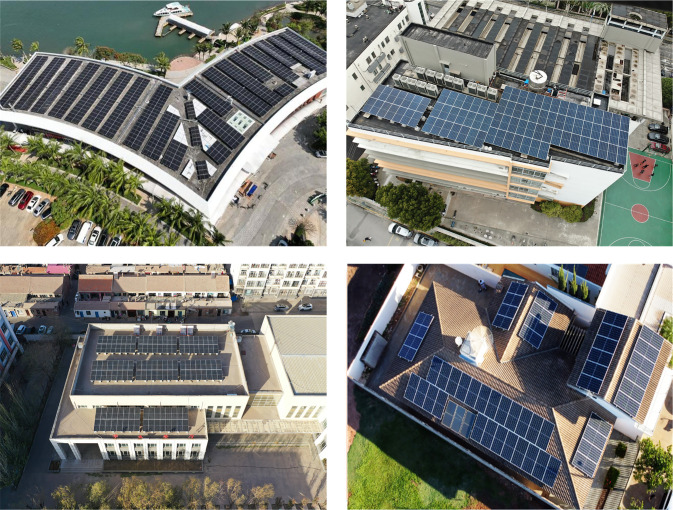
Samples from the independent T2 test set.

Furthermore, we constructed an independent test set (T2). This set allows for a rigorous evaluation of the model’s cross-scenario generalization capabilities. This auxiliary test set contains 1,330 real-world aerial images. These images were sourced from diverse geographic regions and complex environmental scenarios. Representative samples from the main dataset are illustrated in Fig 1. Samples from the T2 test set are shown in Fig 2.

### Proposed method

#### Baseline architecture: YOLOv11.

The YOLO (You Only Look Once) framework is a cornerstone technology. It is fundamental to the field of one-stage object detection. YOLOv11 represents the latest iteration of this series. It builds upon the efficient network architectures of its predecessors, YOLOv8 and YOLOv10. Furthermore, it further optimizes the feature extraction backbone. It also refines gradient propagation paths.

**Fig 3:**
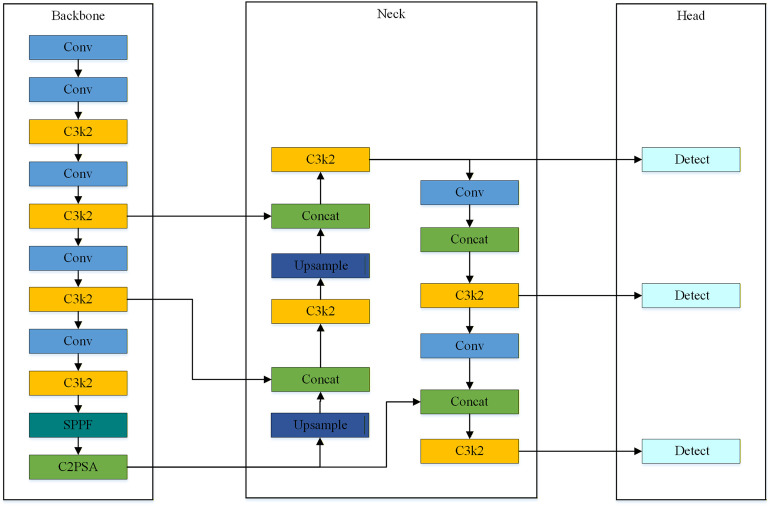
Schematic architecture of the baseline YOLOv11 network.

Fig 3 illustrates the YOLOv11 network architecture. Functionally, it is divided into three core modules. These are the Backbone, the Neck, and the Head. The Backbone adopts an improved Cross Stage Partial (CSP) structure. This design reduces computational complexity. Simultaneously, it ensures rich gradient flow. This enables the effective extraction of deep semantic features. The Neck introduces a Path Aggregation Network (PANet). This facilitates bidirectional multi-scale feature fusion. The Head employs a decoupled design. It processes classification and bounding box regression tasks independently. This strategy mitigates mutual interference between distinct tasks.

YOLOv11 excels in general object detection tasks. However, it faces significant limitations in UAV rooftop PV detection scenarios. In aerial imagery, PV modules typically appear as densely distributed small targets. Their apparent scales fluctuate drastically. This variation corresponds to changes in UAV flight altitude. Furthermore, rooftop scenes contain various distractors. These include shadows, vegetation, and HVAC equipment. Such elements create cluttered backgrounds. Standard convolution operations often struggle in this context. They fail to selectively focus on the discriminative features of PV modules. We propose an improved YOLOv11 model to overcome these limitations. The aim is to achieve an optimal balance between detection accuracy and computational efficiency. Specific improvement schemes are detailed in the following sections.

#### Integration of the P2 Micro-target detection layer.

In the standard YOLOv11 architecture, the feature extraction network typically outputs feature maps at three scales. These are P3, P4, and P5. They correspond to down-sampling factors of 8 × , 16 × , and 32 × , respectively. However, PV modules occupy extremely small pixel areas in rooftop PV detection scenarios. This is especially true when UAVs operate at high altitudes. Consequently, texture and edge information of such small-scale targets is easily lost. This occurs during continuous down-sampling operations. This loss leads to severe missed detection problems.

**Fig 4:**
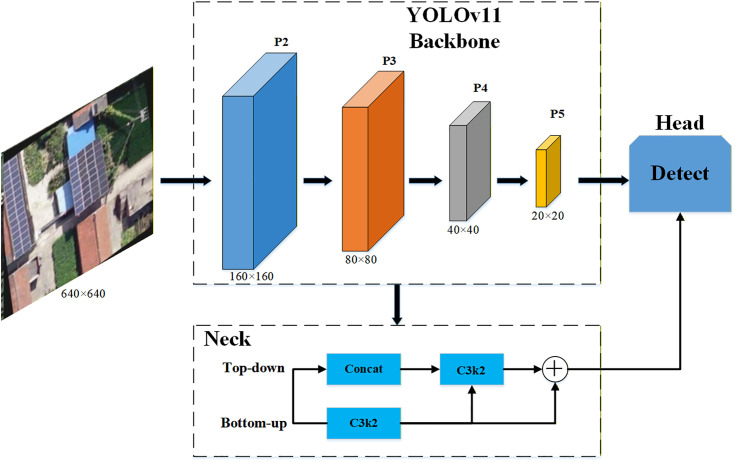
Structural diagram of the improved four-scale feature fusion network.

We introduce a high-resolution P2 detection head to mitigate this challenge [24]. This establishes a four-scale detection framework. Specifically, we utilize shallow high-resolution features from the backbone network. These are from the P2 layer with a 4× down-sampling factor. They are integrated into the neck network via lateral connections. The P2 layer possesses lower semantic information density. However, it preserves rich spatial geometric features. These features are crucial for the precise localization of micro-scale PV targets. We fuse deep semantic information with shallow fine-grained details. As a result, the improved network architecture significantly enhances the model’s perception capability. This is particularly effective for densely distributed PV modules. The improved feature fusion path is illustrated in Fig 4.

#### LSKBlock: Large selective kernel attention module.

The background of rooftop PV power stations is highly complex. PV modules typi-cally present as slender rectangular structures with distinct directional orientations. The standard YOLOv11 algorithm primarily relies on 3 × 3 convolution kernels for feature extraction. However, the receptive field of such kernels is fixed and limited. It is insufficient to capture the global context information of PV arrays. This deficiency causes the model to easily misclassify shadows or background distractors with similar spectral features as targets. Consequently, this leads to a high false detection rate.

**Fig 5:**
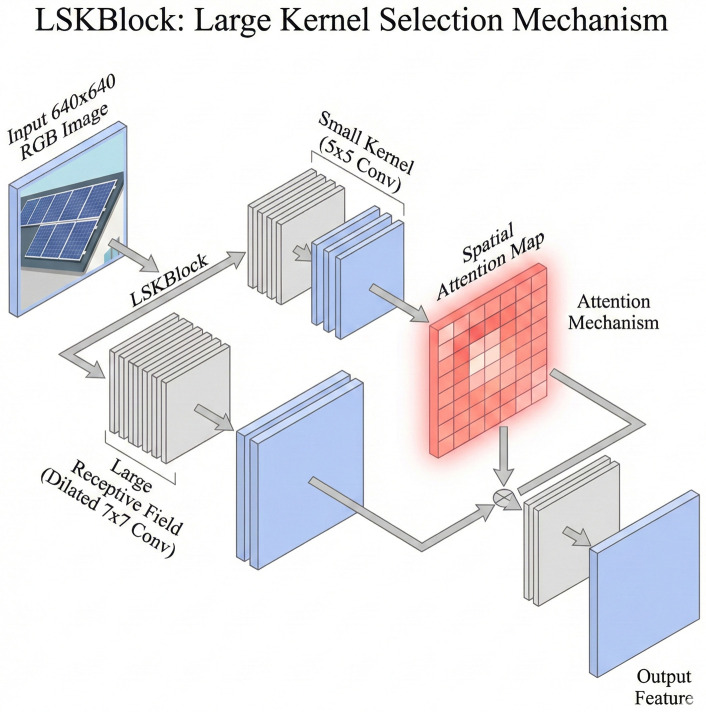
Schematic diagram of the LSKBlock module structure.

To address these issues, we integrated the Large Selective Kernel Attention (LSKBlock) module [25] into the backbone network. The core mechanism of this module lies in its ability to dynamically adjust the receptive field size. This allows it to adapt to the unique features of different targets. As shown in Fig 5, the module decomposes large-scale convolutions. It breaks them down into a sequence of depth-wise convolutions and dilated convolutions. This significantly reduces computational overhead while simultaneously achieving an effective expansion of the receptive field.

The architecture of the LSKBlock consists of two core components. These are the Large Kernel Selection sub-module (LK Selection) and the Feed-Forward Network (FFN). The Large Kernel Selection sub-module decomposes the large receptive field. It uses multiple depth-wise convolutions with progressively increasing dilation rates (specifically, a 5 × 5 standard depth-wise convolution and a 7 × 7 dilated depth-wise convolution). Subsequently, a spatial selection mechanism is employed. This dynamically assigns weights to these multi-scale features. This adaptive mechanism enables the model to selectively focus on discriminative spatial regions. It effectively resolves the inherent problems of drastic target scale variations and environmental noise interference in UAV aerial PV imagery.

#### Lightweight feature extraction via GhostNet module.

The integration of the P2 detection head and the Large Selective Kernel Attention (LSKBlock) significantly improved detection accuracy. However, it also increased the model’s total parameters and computational load. UAV onboard platforms have inherent limitations in computing power and battery life. Therefore, maintaining a lightweight network architecture is crucial. This is essential for meeting real-time operational requirements.

We addressed the parameter surge caused by the P2 layer and LSKBlock. We adopted the lightweight GhostNet module [26] to reconstruct the Backbone and Neck networks. We replaced standard convolutions with Ghost Convolutions (GhostConv). Additionally, we substituted the computationally intensive C3k2 modules with efficient G3Ghost modules. The core advantage of the Ghost module lies in its efficient feature generation. It generates redundant feature maps through linear transformations. This mechanism significantly reduces Floating Point Operations (FLOPs). Notably, this strategy is highly effective in the high-resolution P2 branch (160 × 160 resolution). It reduces computational overhead by approximately 40% to 50%. The reduction of computation in the P2 branch is based on the theoretical property of the Ghost module (s = 2), which approximates a 50% reduction in FLOPs compared to standard convolutions.

**Fig 6:**
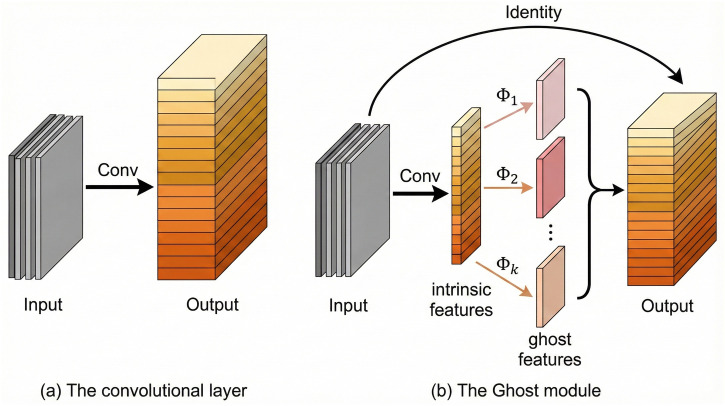
Illustration of the standard convolutional layer and the Ghost module.

As shown in Fig 6, the G3Ghost module first generates a set of intrinsic feature maps. It uses a small number of convolution kernels for this step. Subsequently, low-cost linear operations (such as depth-wise convolution) are performed on these maps. This process generates “ghost feature maps.” Finally, these two sets of feature maps are concatenated to produce the complete feature output. This module significantly compresses model volume while preserving feature representation capabilities. It effectively offsets the computational burden introduced by the P2 layer. Consequently, it achieves an optimal balance between detection accuracy and inference speed.

#### The proposed MS-YOLOv11 architecture.

**Fig 7:**
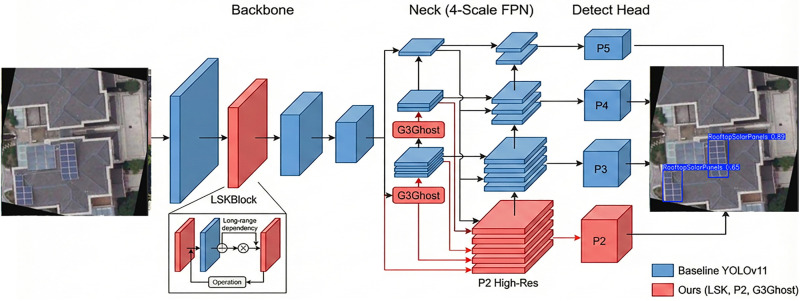
Overall network architecture of the proposed MS-YOLOv11.

This study addresses three synergistic challenges in UAV rooftop PV detection: drastic target scale variations, complex background interference, and limited computational resources. We integrated the previously described improvement strategies into a unified framework. This framework is named MS-YOLOv11 (Multi-Scale YOLOv11). Fig 7 illustrates the complete architecture of the model. MS-YOLOv11 retains the inherent real-time detection advantages of the baseline YOLOv11. Furthermore, it performs deep optimization tailored to UAV remote sensing scenarios. This is achieved through a dual strategy of “Feature Enhancement” and “Cost Reduction and Efficiency Increase.”

We specifically integrated the Large Selective Kernel Attention (LSKBlock) module into the deep stages of the backbone network. This is shown in the bottom-left inset of Fig 7. The module combines spatial selective attention mechanisms with large receptive field convolutions. The LSKBlock is strategically inserted into the **Backbone** network, replacing the standard conv module in **P3**. We chose this specific stage because the P3 layer contains rich spatial details essential for identifying solar panel textures. And the LSKBlock enhances the model’s ability to capture long-range dependencies at this critical scale without significantly increasing the computational burden of the earlier high-resolution stages (P2). The other backbone stages (P2, P4, P5) remain consistent with the baseline YOLOv11 architecture.

Standard YOLOv11 employs a three-scale detection scheme (P3, P4, P5). In contrast, our proposed model reconstructs a four-scale Feature Pyramid Network (4-scale FPN) within the neck network. This prevents information loss for micro-scale targets during high-altitude operations. The model upsamples shallow features and fuses them with deep semantic information. This generates an additional high-resolution P2 layer (160 × 160 resolution). As shown on the right side of the architecture diagram, the new P2 detection head specifically captures PV targets with minimal pixel coverage. This four-scale fusion strategy achieves full-scale target coverage. It ranges from individual micro PV panels to large-scale PV arrays.

The P2 detection head introduces extra computational overhead. To offset this, we drew upon design concepts from GhostNet. We redesigned the feature fusion operators in the neck network using the G3Ghost module. The G3Ghost module replaces standard C3k2 units. It generates redundant feature maps through low-cost linear operations. This design maintains strong feature expression capabilities. Simultaneously, it significantly reduces Floating Point Operations (FLOPs) in the neck network. Ultimately, the model achieves refined four-scale fusion while maintaining high inference speed. This satisfies the requirements for real-time deployment on UAV edge platforms. The primary improvements in this paper are highlighted in the red regions of the architecture diagram.

## Results and discussion

### Experimental platform and hyperparameter settings

To ensure the fairness and comparability of the experimental results, all model training and testing procedures were conducted on a unified hardware and software platform. The hardware environment was based on a high-performance workstation equipped with an Intel(R) Core(TM) i7-14700KF (3.40 GHz) processor and an NVIDIA GeForce RTX 4080 Ti GPU (dedicated memory utilized for accelerating deep learning convolutional operations).

Regarding the software environment, the Windows 10 operating system was employed. The computational environment was managed via the Anaconda platform using Python 3.9.0. The deep learning models were implemented using the PyTorch 1.8.0 framework, integrated with the CUDA 12.4 computing architecture and the corresponding cuDNN acceleration library to optimize training efficiency.

To maintain consistency during the optimization of the proposed MS-YOLOv11 and other baseline models, a standardized set of training hyperparameters was applied. The specific network parameter configurations are detailed in Table 1.

**Table 1:** Network Parameter Configurations.

Network parameter	Set value
epoch	300
batch	15
image size	640*640
learing rate	0.01
momentum	0.937
training ratio	9:1
iou	0.7
Workers	4

### Evaluation metrics

To objectively and quantitatively evaluate the detection performance of the pro-posed MS-YOLOv11 on rooftop PV panels, this study selects Precision (P), Recall (R), Average Precision (AP), and Model Parameters (Ps) as the primary evaluation metrics.

P: Measures the accuracy of the model’s predictions by calculating the proportion of true PV panels among all detected targets. R: Quantifies the model’s retrieval capability, representing the ratio of ground-truth PV panels correctly identified by the model. AP: Defined as the area under the Precision-Recall (P-R) curve, this metric provides a comprehensive assessment of the model’s detection performance across various confidence thresholds. In multi-class detection tasks, the mAP is the arithmetic mean of AP values across all categories. Since this study focuses exclusively on “rooftop PV panels” as a single target category (m = 1), the mAP value is numerically identical to the AP. Additionally, Model Parameters (Ps) is utilized to evaluate the lightweight characteristics and memory footprint of the architecture following the proposed modifications.

The mathematical expressions for these metrics are defined as follows:


$$P=\frac{TP}{TP+FP}\times 100\%$$ (2)

$$R=\frac{TP}{TP+FN}\times 100\%$$ (3)

$$AP=\overset{1}{\underset{0}{\int }}P\left(R\right)\text{dR}\times \text{100\textbackslash{}\%}$$ (4)

Where TP (True Positives) denotes the number of PV panels correctly detected; FP (False Positives) refers to the number of background objects incorrectly identified as PV panels; and FN (False Negatives) represents the number of ground-truth PV panels missed by the model.

### Loss function

The loss function adopted by YOLOv11 in this study consists of three components: Classification Loss, Box Regression Loss, and Distribution Focal Loss. The expression for the total loss function (LOSS) is given as follows:


$$L_{total}=\lambda_{box}L_{box}+\lambda_{cls}L_{cls}+\lambda_{dfl}L_{dfl}$$ (5)

Where, L_cls_ measures the model’s ability to correctly predict the target category (i.e., photovoltaic panel or background in this study). L_box_ quantifies the geometric overlap between the Prediction Box and the Ground Truth Box, with CIoU Loss (Complete IoU) used as the default metric here. L_dfl_ is employed to optimize the boundaries of regression boxes and address the issue of ambiguous detection boxes. All hyperparameters are set according to the official configuration of YOLOv11.

Nevertheless, the optimization and improvement of the loss function merit further exploration. For the task of UAV-based rooftop photovoltaic panel detection, subsequent optimization of the loss function is highly applicable. Considering the unique characteristics of photovoltaic panel detection, shifting the focus from geometric accuracy to decision-driven efficiency represents a promising direction for future research [27].

### Training performance and convergence of the baseline YOLOv11 model

**Fig 8:**
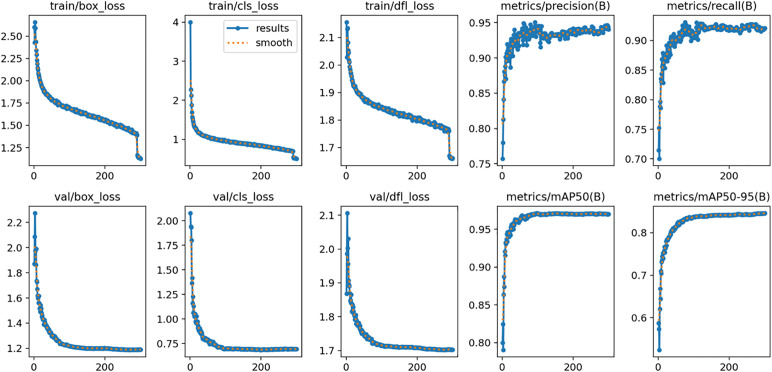
Training dynamics of the baseline YOLOv11 model.

We monitored the trends of loss functions and performance metrics during training. This was done to evaluate the learning effect of the baseline YOLOv11 model on our constructed rooftop PV dataset. Relevant results are presented in Fig 8.

The loss function curves reveal specific trends. Both bounding box regression loss and classification loss for training and validation sets decreased rapidly. This monotonic decline occurred within the initial 50 epochs. This phenomenon indicates excellent performance by the YOLOv11 architecture. It excels in extracting discriminative features. It also achieves rapid model convergence in the early stages. The loss curves gradually stabilized after approximately 100 epochs. They subsequently entered a plateau phase. Notably, the validation set loss consistently tracked the downward trend of the training set loss. No significant deviation or rebound occurred. This demonstrates the baseline model’s strong fitting capability under the current hyperparameter configuration. It effectively avoids overfitting issues.

Regarding detection performance metrics, the model showed positive trends as training advanced. P, R, and AP all ex-hibited a steady increase. Ultimately, the baseline YOLOv11 model achieved a P of 93.76% and a R of 92.55%. AP at an Intersection over Union (IoU) threshold of 0.5 (mAP@0.5) was approximately 97%. The AP across the 0.5 to 0.95 IoU interval (mAP@0.5:0.95) reached 85%. In this study, the evaluation metrics, specifically mAP@0.5 and mAP@0.5:0.95, are calculated strictly following the standard **Microsoft COCO evaluation protocol**. Here, mAP@0.5 represents the mean average precision at an Intersection over Union (IoU) threshold of 0.5, while mAP@0.5:0.95 represents the average mAP over 10 different IoU thresholds ranging from 0.5 to 0.95 with a step size of 0.05.

The baseline model has demonstrated competitive detection accuracy. However, in-depth analysis of training dynamics reveals room for further optimization. This is particularly relevant for high-precision localization tasks in complex background envi-ronments. Continuous fluctuations appeared in the Precision and Recall curves during the convergence phase. This reflects potential instability in the model. Specifically, it struggles when processing targets with extreme scale variations. These findings provide a rigorous benchmark reference. They serve as an empirical basis for formulating subsequent optimization strategies.

### Training performance and convergence of the proposed MS-YOLOv11

**Fig 9:**
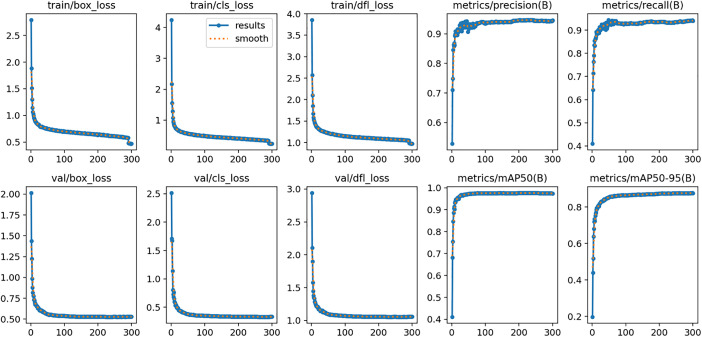
Training and convergence performance of the proposed MS-YOLOv11 model.

To verify the efficacy of the proposed MS-YOLOv11 model, the dynamic evolution of its loss functions and performance metrics throughout the training phase was metic-ulously recorded, as illustrated in Fig 9. Overall, the model demonstrates exceptional convergence speed and superior training stability.

As observed from the loss trajectories, the box regression loss, classification loss (Cls loss), and distribution focal loss (Dfl loss) exhibited a sharp monotonic decline during the initial training stage (the first 50 epochs). This rapid descent indicates that the enhanced feature extraction backbone of MS-YOLOv11 can swiftly capture discriminative object features, significantly accelerating gradient descent efficiency. By approximately the 100th epoch, the loss curves for both the training and validation sets gradually plateaued at a low level. The validation loss remained consistently synchronized with the training loss without any rebounding, thereby demonstrating the model’s robust generalization capability and its resistance to overfitting.

Regarding detection performance, the P, R, and mAP values showed a steady and synchronized ascent with the increase in training epochs. Ultimately, the MS-YOLOv11 model achieved a Precision of 96.32%, a Recall of 95.74%, and an mAP@0.5 of 98.5%, with the mAP@0.5:0.95 stabilizing at approximately 88.8%.

Notably, the high mAP@0.5:0.95 score underscores the superior capability of MS-YOLOv11 not only in accurate target classification but also in generating high-quality bounding boxes that align closely with ground-truth annotations. This validates the significant advantages of the multi-scale feature fusion strategy and the LSKBlock in enhancing localization precision. For clarity, mAP@0.5 primarily assesses the model’s detection capability at a standard overlap threshold, while mAP@0.5:0.95 (calculated across IoU thresholds from 0.5 to 0.95 with a step of 0.05) reflects the model’s robustness in high-precision localization and spatial overlap accuracy.

### Qualitative analysis and visualization of detection results

**Fig 10:**
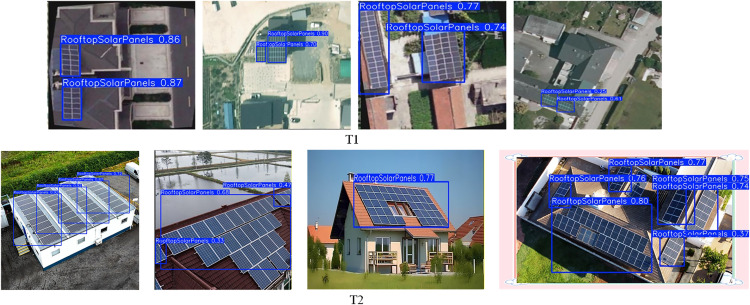
Visualization of detection performance.

We conducted visualization experiments based on two independent test sets. This was done to visually evaluate the robustness and generalization capabilities of the pro-posed MS-YOLOv11 model in complex real-world scenes. Relevant results are presented in Fig 10. Specifically, the T1 test set is an independent subset derived from the original dataset. It primarily contains standard satellite imagery and high-altitude aerial imagery. In contrast, the T2 test set was independently constructed. Its data consists of low-altitude UAV imagery collected under varying viewing angles, altitudes, and lighting conditions.

Detection results from the T1 test set demonstrate the model’s performance. It ex-hibits extremely high confidence on source domain data. MS-YOLOv11 achieves high-precision detection even against complex urban textures and severe shadow interference. Additionally, the improved network effectively eliminates missed and false detections in scenes with densely distributed small targets. This performance fully validates the model’s capability. It possesses excellent fine-grained spatial information retention during the feature extraction stage.

Detection results from the T2 test set highlight the model’s robust generalization performance. T2 imagery exhibits significant domain shift phenomena. These include transitions from nadir to oblique viewing angles and drastic variations in target scale. Despite this, MS-YOLOv11 maintains stable and reliable detection efficacy. The model performs well even in high-interference environments. Examples include backgrounds adjacent to water bodies or close-range large-scale target scenarios. In these cases, predicted bounding boxes align precisely with target edges. Model confidence showed a minor decline in some samples with large inclination angles or low contrast. However, no significant missed detections occurred. These results indicate that MS-YOLOv11 does not merely fit the training data distribution. Instead, it truly learns the inherent geometric and semantic features of PV panels. This confirms its potential for deployment in dynamic, variable, and unpredictable real-world field environments.

## Discussion

### Comparison with State-of-the-Art methods

We rigorously evaluated the detection efficacy and operational efficiency of the YOLOv11 framework. To do this, we selected representative mainstream algorithms from the object detection field for comparative analysis. The selected baseline models include the two-stage detector Faster R-CNN. We also included the one-stage detector SSD [28]. Furthermore, we incorporated subsequent iterations of the YOLO series. These are YOLOv5n, YOLOv8n, and YOLOv10n. All baseline models were trained using their official recommended hyperparameters. Initial learning rate, weight decay, and mosaic augmentation probabilities as provided in their respective Ultralytics/official repositories. The only shared setting was the input resolution (640 × 640) and the total number of epochs to ensure consistent convergence conditions.

Table 2 reveals the performance data. The YOLOv11n model achieved a mean Av-erage Precision at mAP@0.5 of 97.1%. This represents a significant improvement over Faster R-CNN and SSD. The gains are 7.95% and 15.8%, respectively. Furthermore, this model outperforms other YOLO series variants. This fully demonstrates its superiority in comprehensive detection capabilities. Notably, YOLOv11n achieved a value of 85.0% on more stringent metrics. These metrics strictly evaluate localization precision. This indicates the model possesses excellent robustness and regression accuracy. It handles complex environmental factors effectively. Examples include PV panel boundary overlap and shadow interference.

**Table 2:** Performance comparison of different target detection models on the rooftop PV dataset.

Model	P(%)	R(%)	mAP@0.5 (%)	mAP@0.5:0.95 (%)	Params (M)
Faster R-CNN	85.24	88.4	89.15	62.3	41.53
SSD	82.1	79.55	81.3	55.45	24.3
YOLOv5n	88.35	86.12	89.5	70.15	1.9
YOLOv8n	90.12	89.45	92.3	75.8	3.2
YOLOv10n	92.5	91.2	95.8	82.1	2.3
YOLO11n	93.76	92.55	97.1	85	2.59

YOLOv11n exhibits a superior level of architectural optimization regarding struc-tural efficiency. The model comprises only 2.59 million parameters. This is significantly lower than Faster R-CNN’s 41.53 million. It is also lower than SSD’s 24.3 million. Despite this, it consistently outperforms both on all precision metrics. YOLOv5n maintains the smallest model volume with 1.9 million parameters. However, its mAP@0.5:0.95 metric is 14.85% lower than that of YOLOv11n. The data above fully substantiates the efficiency of the YOLOv11 architecture. It optimizes convolution operators and feature extraction modules. Consequently, it achieves a substantial performance leap at minimal computational cost. This characteristic renders it an ideal deployment model. It is well-suited for resource-constrained UAV embedded platforms.

Experimental data reveal a steady upward trend in model precision from YOLOv8 to YOLOv11. We considered the specific characteristics of UAV PV detection tasks. These include variable target scales, high distribution density, and cluttered backgrounds. YOLOv11n achieved a balanced performance in this context. It attained a P of 93.76% and a R of 92.55%. This balance underscores the inherent advantages of its backbone network. It excels in capturing fine-grained features. Consequently, our experimental results provide a high-performance benchmark reference for subsequent work. They also suggest implications for the multi-scale feature fusion module introduced later. This module will further overcome performance bottlenecks in extreme small object detection tasks.

### Ablation study and synergy analysis

We conducted a series of ablation studies to systematically evaluate the contribution of each proposed improvement strategy to rooftop PV detection performance. We used YOLOv11n as the baseline model. As shown in Table 3, we designed five different experimental configurations. These involved progressively integrating the P2 layer, the LSKBlock module, and the G3Ghost module.

**Table 3:** Ablation study of the proposed MS-YOLOv11 on the rooftop PV dataset.

Group	Baseline	P2 Layer	LSKBlock	G3Ghost	P (%)	R (%)	mAP@0.5 (%)	mAP@0.5:0.95 (%)	Params (M)	Pt (M)
1	✓				93.76	92.55	97.1	85	2.59	5.37
2	✓	✓			94.25	94.17	97.8	86.96	2.65	5.5
3	✓		✓		95.42	93.24	98.2	87.68	2.58	5.29
4	✓	✓	✓		96.05	95.35	98.4	88.4	2.87	6.11
5 (Ours)	✓	✓	✓	✓	96.32	95.74	98.5	88.8	2.04	4.5

Table 3 reveals the results. Group 1 represents the baseline model. It achieved an mAP@0.5 of 97.1%. Group 2 is the model integrated only with the P2 layer. Its mAP@0.5 improved to 97.8%. Additionally, Recall (R) significantly increased by 1.62%. This performance gain validates the effectiveness of the high-resolution P2 branch. This branch effectively retains fine-grained spatial information. Such information is essential for identifying micro-scale PV panels in high-altitude UAV imagery. However, this network expansion also increased the computational burden. The model volume grew from 5.37 MB to 5.50 MB.

Similarly, Group 3 is the model embedded only with the LSKBlock module. It reached an mAP@0.5 of 98.2%. This confirms the module’s significant role. It excels in capturing long-range spatial dependencies and suppressing complex background interference. Group 4 integrates both the P2 layer and the LSKBlock module. The model’s Precision (P) reached 96.05%, and mAP@0.5 reached 98.4%. This performance leap highlights the synergistic effect of the two improvement strategies. The P2 layer provides the necessary resolution support for detection tasks. Meanwhile, the LSKBlock module optimizes and refines the extracted features through spatial attention mechanisms.

Group 4 achieved improved detection accuracy. However, its parameter count peaked at 2.87 million. The model volume increased to 6.11 MB. This poses a challenge for deployment on resource-constrained UAV edge platforms. We proposed the final architecture, MS-YOLOv11, to address this issue. Group 5 further integrated the G3Ghost module. Experimental results show that the Group 5 model outperformed all other configurations across all metrics. Precision reached 96.32%. Recall reached 95.74%. mAP@0.5 reached 98.5%.

Most notably, the G3Ghost module significantly reduced the network’s structural complexity. It achieved this by reconstructing feature redundancy. Compared to the Group 4 model, MS-YOLOv11 reduced the parameter count by approximately 29%. The model volume was also compressed to 4.5 MB.

In summary, the ablation study results demonstrate a key finding. The improved architecture proposed in this study achieves an optimal balance between detection sensitivity and computational efficiency. Ultimately, compared to the baseline YOLOv11n, MS-YOLOv11 showed significant improvements. Precision, Recall, and mAP@0.5:0.95 increased by 2.56%, 3.19%, and 3.8%, respectively. Simultaneously, the parameter count decreased by 0.55 million. The total model volume was reduced by 0.87 MB.

### Comparative Visualization of Detection Results

#### Detection results based on the T1.

**Fig 11:**
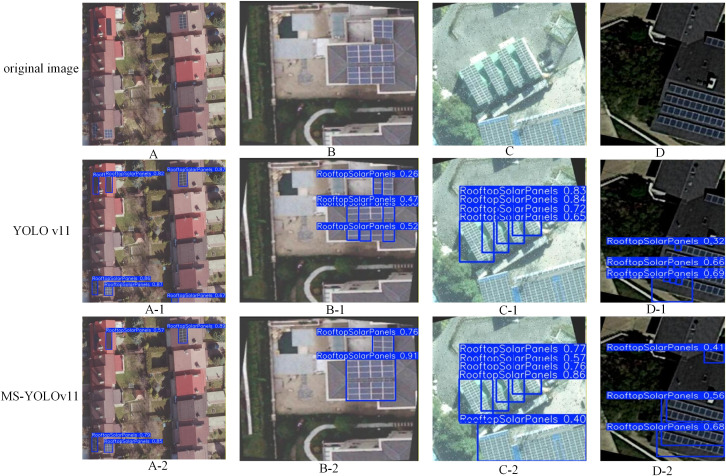
Detection results of the test set T1.

To intuitively demonstrate the effectiveness of the proposed modules, a qualitative comparison between the baseline YOLOv11 and our MS-YOLOv11 on the T1 was presented in Fig 11. The selected scenes encompass challenging conditions typical of UAV imagery, including dense arrangements, severe occlusions, and complex backgrounds.

It can be seen from the detection figures that B-1, the baseline YOLOv11 generates multiple redundant and poorly localized boxes for a single, contiguous PV array. In sharp contrast, B-2 demonstrates that MS-YOLOv11 entirely resolves this issue, outputting a clean, highly accurate, and tightly fitted bounding box. This visual improvement directly validates the theoretical advantage of the LSKBlock. By dynamically adjusting the spatial receptive field, the LSKBlock selectively focuses on the true boundaries of the target while suppressing confusing intra-array edge features. This mechanism effectively eliminates redundant detections and tightens the bounding boxes, perfectly corroborating the significant quantitative boost observed in our mAP@0.5:0.95 metrics.

Furthermore, the baseline model is highly susceptible to missed detections when panels are occluded by shadows or located at the image periphery. As shown in C-1, YOLOv11 completely fails to detect the large panel array at the bottom of the image due to poor contrast. Similarly, in D-1, the baseline misses the small panels at the top right and the heavily shadowed panels at the bottom. However, our MS-YOLOv11 successfully retrieves these challenging targets (C-2 and D-2). This robust recall capability is primarily attributed to the integration of the high-resolution P2 layer. By preserving fine-grained textural details that are typically lost in deeper layers, the P2 layer provides the essential discriminative features needed to identify small or low-contrast targets. This visual evidence strongly supports our statistical findings (Table 4), where the introduction of the P2 layer led to a substantial reduction in False Negatives.

**Table 4:** Statistical results of the detection data.

Model	P(%)	R(%)	TP	FP	FN	Objective
**1. Baseline**	**93.76%**	**92.55%**	**4787**	**319**	**385**	**5172**
**2. + P2 Layer**	**94.25%**	**94.17%**	**4870**	**297**	**302**	**5172**
**3. + LSKBlock**	**95.42%**	**93.24%**	**4822**	**231**	**350**	**5172**
**4. + P2 + LSK**	**96.05%**	**95.35%**	**4932**	**203**	**240**	**5172**
**5. Ours (Full)**	**96.32%**	**95.74%**	**4952**	**189**	**220**	**5172**

#### Detection results based on the T2.

**Fig 12:**
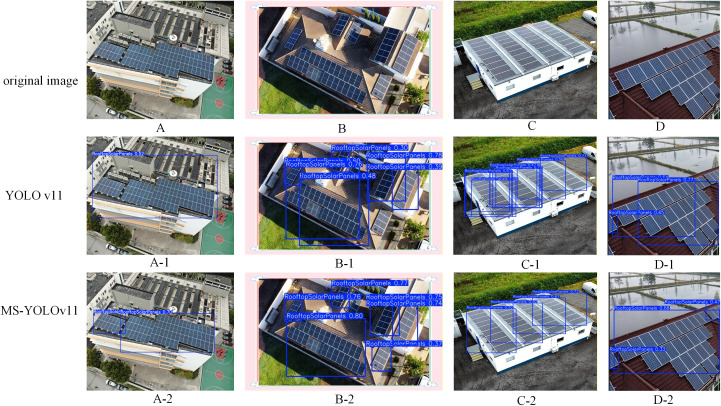
Detection results of the test set T2.

To further validate the generalization capability and robustness of the proposed MS-YOLOv11 across diverse and novel environments. We conducted supplementary evaluations on an independent test dataset, T2. As illustrated in Fig 12, this dataset features varying UAV flight altitudes, distinct architectural styles, and different illumination conditions, posing a significant challenge to the detector’s adaptability.

When exposed to novel scenes, the baseline YOLOv11 exhibits severe feature dilation, leading to excessively oversized bounding boxes. For example, in A-1 and D-1, the baseline model fails to delineate the boundaries of the PV arrays accurately, generating massive boxes that inappropriately encapsulate large areas of irrelevant background, such as roof structures and air conditioning units. This indicates a lack of spatial adaptability in the baseline’s attention mechanism. Conversely, A-2 and D-2 demonstrate that MS-YOLOv11 effectively restrains this dilation. Driven by the LSK module, our model dynamically adjusts its receptive field to match the exact scale of the targets, consistently outputting tight and precise bounding boxes even in unfamiliar scenes. This visual evidence firmly establishes the cross-domain robustness of the LSKBlock in solving the “oversized box” dilemma.

Furthermore, the baseline model struggles with contiguous PV arrays in the supplementary dataset. It produces chaotic, overlapping, and fragmented predictions. As seen in B-1 and C-1, YOLOv11 generates multiple redundant bounding boxes for the same array sections. This flaw reflects a poor understanding of global context. In stark contrast, MS-YOLOv11 resolves this issue. As shown in B-2 and C-2, it outputs clean, distinct, and logically separated bounding boxes. This success highlights a key advantage of our model. The P2 layer provides high-resolution texture extraction, while the LSKBlock ensures contextual boundary suppression. Together, this synergy proves to be highly generalizable across new environments.

In summary, the exceptional performance on the supplementary T2 dataset verifies that MS-YOLOv11 does not merely overfit to the primary training data. Instead, it exhibits strong robustness and generalization, ensuring reliable and high-precision PV panel detection in unpredictable, real-world UAV inspection scenarios.

#### Statistical analysis of detection results.

As shown in Table 4, we evaluated 1,300 test images containing 5,172 PV panel targets. The results demonstrate that the addition of the P2 layer successfully reduced both FN and FP counts. Theoretically, introducing high-resolution feature layers (P2) is often associated with the risk of introducing background noise, which could potentially lead to an increase in false detections. In practice, however, the P2 layer provided critical textural details that allowed the detector to better separate ambiguous background artifacts from true panels, leading to higher precision.

The implementation of the LSKBlock further reduced False Positives by 88. Although False Negatives also dropped by 35, the gain in Recall was not as substantial as with the P2 layer alone. The attention mechanism naturally tends to expand the bounding boxes. Yet, in the context of high-resolution imagery, this slight dilation has a negligible impact on the final outcome. Crucially, the LSKBlock captures global context, allowing the model to reject isolated background artifacts and filter out hard negatives.

By combining the advantages of these two modules, the model achieves superior performance, as evidenced by both the visual outputs and the statistical data. Ultimately, paired with a lightweight architecture, MS-YOLO is well-suited for the task of UAV-based rooftop PV inspection.

## Conclusion

This study proposes MS-YOLOv11, an improved lightweight and high-performance detection model. It aims to address three core challenges inherent in UAV rooftop PV inspection. These are drastic target scale variations, complex background interference, and limited computational resources.

The integration of a high-resolution P2 detection layer effectively preserves shallow fine-grained spatial geometric features. This significantly improves localization precision for dense and micro-scale targets. Ablation study results indicate significant improvements compared to the baseline model. The introduction of the P2 layer increased R by 1.62%. It also resulted in substantial growth in mAP. We embedded the LSKBlock module. This allows the model to dynamically adjust its receptive field to capture long-range spatial dependencies. Consequently, it robustly distinguishes PV arrays amidst complex rooftop backgrounds. Experimental data reveals the impact of the LSKBlock module. It increased the mAP at an IoU threshold of 0.5 from 97.1% to 98.2%. Furthermore, we introduced the G3Ghost module to reconstruct the network via linear transformations. This successfully offsets the computational overhead brought by the multi-scale architecture. It also achieves lightweight model compression.

The proposed MS-YOLOv11 model outperforms the baseline YOLOv11n across core metrics. These include P, R and mAP. Simultaneously, it reduces parameter count by 0.55 million. The total model volume is compressed by 0.87 MB. The model contains only 2.04 million parameters. Its size is a mere 4.50 MB. The inference speed of 115 FPS was evaluated on an NVIDIA RTX 3060 GPU with a batch size of 1, corresponding to a latency of 8.7 ms per image. It meets the rigorous requirements for real-time deployment. Qualitative results from the cross-domain T2 further confirm the model’s capabilities. It possesses robust generalization across different viewing angles and lighting conditions. In summary, MS-YOLOv11 provides a reliable and efficient technical solution. It enables autonomous UAV PV inspection on resource-constrained edge devices.
